# Parkin depletion delays motor decline dose-dependently without overtly affecting neuropathology in α-synuclein transgenic mice

**DOI:** 10.1186/1471-2202-14-135

**Published:** 2013-11-05

**Authors:** Margot Fournier, Amandine Roux, Jérôme Garrigue, Marie-Paule Muriel, Paul Blanche, Hilal A Lashuel, John P Anderson, Robin Barbour, Jiping Huang, Sophie Tezenas du Montcel, Alexis Brice, Olga Corti

**Affiliations:** 1 Inserm, U 975, CRICM, Hôpital de la Pitié-Salpêtrière, F-75013 Paris, France; 2 UPMC Université Paris 06, UMR_S975, F-75013, Paris, France; 3 CNRS, UMR 7225, F-75013, Paris, France; 4 Laboratory of Molecular Neurobiology and Chemical Biology of Neurodegeneration, Brain Mind Institute, Ecole Polytechnique Fédérale de Lausanne, Lausanne CH-1015 Switzerland; 5 Université Pierre et Marie Curie-Paris 6, ER4 Modeling in Clinical Research, Paris F-75013 France; 6 Elan Pharmaceuticals, 180 Oyster Point Blvd, South San Francisco, California, CA 94080 USA; 7 Department of Biostatistics and Medical Informatics, Hôpital de la Pitié-Salpêtrière, Assistance Publique-Hôpitaux, 47 Boulevard de l'Hôpital, Paris F-75013 France; 8 AP-HP, Hôpital de la Salpêtrière, Department of Genetics and Cytogenetics, F-75013 Paris, France; 9 Current address: Center for Psychiatric Neuroscience, Department of Psychiatry, Lausanne University Hospital, Prilly-Lausanne, CH 1008 Switzerland

**Keywords:** α-syn phosphorylation, α-syn truncation, Ubiquitin, Posttranslational modifications, Transgenic mice overproducing α-syn, *parkin* knockout mice, Parkinson’s disease

## Abstract

**Background:**

Mutations of the gene encoding the major component of Lewy bodies (LB), α-synuclein (α-syn), cause autosomal dominant forms of Parkinson’s disease (PD), whereas loss-of-function mutations of the gene encoding the multifunctional E3 ubiquitin-protein ligase Parkin account for autosomal recessive forms of the disease. Parkin overproduction protects against α-syn-dependent neurodegeneration in various *in vitro* and *in vivo* models, but it remains unclear whether this process is affected by Parkin deficiency. We addressed this issue, by carrying out more detailed analyses of transgenic mice overproducing the A30P variant of human α-syn (hA30Pα-syn) and with two, one or no *parkin* knockout alleles.

**Results:**

Longitudinal behavioral follow-up of these mice indicated that Parkin depletion delayed disease-predictive sensorimotor impairment due to α-syn accumulation, in a dose-dependent fashion. At the end stage of the disease, neuronal deposits containing fibrillar α-syn species phosphorylated at S129 (P^S129^α-syn) were the predominant neuropathological feature in hA30Pα-syn mice, regardless of their *parkin* expression. Some of these deposits colocalized with the LB markers ubiquitin and α-syn truncated at D135 (α-synD135), indicating that P^S129^α-syn is subjected to secondary posttranslational modification (PTM); these features were not significantly affected by *parkin* dysfunction.

**Conclusions:**

These findings suggest that Parkin deficiency acts as a protective modifier in α-syn-dependent neurodegeneration, without overtly affecting the composition and characteristics of α-syn deposits in end-stage disease.

## Background

Biochemical studies of α-syn within Lewy bodies (LB) revealed various posttranslational modifications (PTM) of the protein, some of which are associated with Parkinson’s disease (PD) and other synucleinopathies (for a recent review on α-syn PTM, see [1]). The most abundant α-syn PTM is phosphorylation: nearly 90% of the molecules of this protein in LB are phosphorylated at S129 (P^S129^α-syn) [2,3]. α-Syn is also ubiquitylated [2,4-6]; various lysine residues affected by this PTM have been identified, mostly in the N-terminal part of the protein [2,7,8]. Ubiquitin has been shown to be primarily associated with P^S129^α-syn [2,4,5], raising the possibility of crosstalk between the two modifications. In addition, about 15% of α-syn is truncated in LB [9,10] and various C-terminally cleaved α-syn species (e.g. D135, Y133, N122, D119, D115) have been identified [2,4,9-12]. All these α-syn species have been shown to accumulate in animal models of α-synucleinopathy [11,13-24], but they have mostly been studied independently of each other. Thus, the interplay between them remains poorly understood.

Most early-onset autosomal recessive cases of PD are due to mutations in the gene encoding Parkin [25,26], an E3 ubiquitin-protein ligase involved in the ubiquitylation of a number of substrates (e.g. CDCRel-1, Pael R, synphilin, p38/JTV1/AIMP2, Eps15) and in various cellular mechanisms, including signaling pathways, synaptic transmission, proteasomal protein degradation and the autophagy of dysfunctional mitochondria [25,27]. It is unclear whether mutations in the *α-syn* and *parkin* genes converge into common neuropathological pathways. The overproduction of Parkin protects against α-syn-induced toxicity, both *in vitro* and *in vivo*[19,28-30], but conflicting results have been reported in models of Parkin depletion, which mimics the loss of protein function underlying PD due to *parkin* mutations [16,31].

We explored this issue further, in mice producing the pathogenic human A30P variant of α-syn (hA30Pα-syn). Transgenic hA30Pα-syn mice are a well described model of α-synucleinopathy, characterized by an age-dependent lethal movement disorder associated with the deposition of proteinase K-resistant α-syn, P^S129^α-syn and ubiquitin throughout the central nervous system [16,21,32]. We previously reported that Parkin deficiency due to biallelic gene knockout delays the onset of the neurodegenerative phenotype in this model [16]. In this study, we used a set of behavioral tests to monitor the development of motor dysfunction in hA30Pα-syn mice with two, one or no functional *parkin* alleles. We also provide a detailed immunohistochemical description of the α-syn deposits, to clarify the relationships between the various PTM of the protein and the effects of an absence of Parkin. We report that dysfunctional *parkin* alleles delay the onset of disease signs, in a dose-dependent manner, in hA30Pα-syn mice, with only modest effects on neuropathological characteristics in end-stage disease.

## Methods

### Ethics statement

All experiments involving mice were approved by the Ile-de-France Regional Ethics Committee for Animal Experiments, Nu3 (P3/2006/006). *Post-mortem* samples of PD patients were obtained from brains collected in a Brain Donation Program of the Brain Bank “GIE NeuroCEB”, run by a consortium of Patient Associations: ARSEP (association for research on multiple sclerosis), CSC (cerebellar ataxias), *France Alzheimer* and *France Parkinson*. Consents were signed by the patients or their next of kin in their name, in accordance with French bioethics law. The approval for the collection of samples in the Brain Bank has been granted by the Ministry of Higher Education and Research (authorization #AC 2007–5).

### Behavioral analyses

Experimental groups of female littermate mice were generated, genotyped and housed, as previously described [16]. In brief, homozygous *parkin* knockout mice [33] brought into the C57Bl/6j genetic background by an accelerated backcross procedure were bred with homozygous hA30Pα-syn mice brought into a C57Bl/6j background by eight consecutive backcrosses [21]. Mice of the double-heterozygous generation were then crossed to generate littermates of the nine expected genotypes. Age-matched littermates of the genotypes of interest were used for all subsequent analyses. Behavioral studies were performed in the mornings of two consecutive days, by investigators blind to genotype. The animals were weighed, and behavioral tasks were performed in the following order: 1) extension reflex, 2) rotarod test and 3) footprint test. The symmetry of the hindlimb extension reflex and the time spent on a rotating rod were studied as previously described [16]. The amplitude of the extension reflex was characterized as follows: 5- swift and wide, 4- normal, 3- mild, 2- narrow, 1- little movement 0- no extension. On this scale, 3 was considered to correspond to an abnormally low score, because it was not observed in control groups; a score of 2 or 1 was invariably accompanied by an abnormal gait. For the footprint test, the hindpaws and forepaws were inked with different colors and the animal was placed at the entrance of a corridor (10 cm long x 5 cm wide x 5 cm high) covered with a paper sheet; the mouse was allowed to walk through without pause. Based on consecutive imprints, we measured the length and width of four steps for the forelimbs and hindlimbs.

### Qualitative and quantitative immunofluorescence analyses

Three cases of sporadic PD and an age-matched control without neurological lesions were selected from the Escourolle Neuropathology Laboratory of Pitié-Salpêtrière Hospital. The PD cases were characterized histopathologically for neuron loss from the *substantia nigra* and for the presence of LB, as previously described [34]. All histological immunofluorescence staining was performed as described by Vitte *et al*. [34], after bleaching to eliminate the autofluorescence of the human tissue, by incubating the sections in phosphate-buffered saline (PBS) for 2 h under the light of a desk lamp at 4°C. All immunohistochemical analyses in animals were performed in homozygous hA30Pα-syn+/+ mice with (*parkin*-/-) or without Parkin deficiency. Animals were killed in the end stage of the disease, about three weeks after the onset of symptoms, and the brain was removed and processed for immunohistochemistry, as previously described [16]. For preabsorption experiments, an antibody directed against α-syn truncated at D135 (α-synD135) was first incubated for 6 h in PBS supplemented with 4% bovine serum albumin (BSA), 10% newborn goat serum (NGS) with the recombinant proteins of interest (α-synD135, α-synY133 and α-syn), at an antibody:protein ratio of 1:2000. The proportion of LB detected by P^S129^α-syn staining also displaying α-synD135 labeling was estimated on 50 LB from the three cases of PD. The percentage of cell bodies and processes costained for the different markers was quantified in two or three representative 5 μm-thick coronal brainstem sections selected on the basis of staining abundance and observed with an epifluorescence microscope (magnification x63, Axioplan 2, Zeiss, Germany). We scored about 50 neuronal cell bodies and 100 processes, identified on the basis of morphology and DAPI staining, for each animal, and six to ten animals from each genotype were analyzed. Brightfield images were acquired with a multipurpose zoom microscope (NIKON AZ100) and NIS software. Colocalization of different types of immunofluorescent labeling was further analyzed with a confocal microscope (Leica SP2 AOBS, Wetzlar, Germany) and a x63 objective. Z-stack images, with 0.2 μm Z-intervals covering entire immunostained volumes within individual cell bodies or processes (*n* = 6 structures), were taken from representative animals (*n* = 3) for each relevant genotype. The degree of colocalization was determined by calculating Pearson’s coefficient with the JACoP plug-in of ImageJ software.

### Immunostaining for electron microscopy

Mice were anesthetized with pentobarbital (130 mg/kg, i.p.; Sigma, St Quentin, France) and perfused transcardially with 4% PFA freshly prepared in PBS supplemented with 0.01% glutaraldehyde. Brains were removed and post-fixed by incubation overnight in 4% PFA. Sections from the brainstem (45 μm thick), which has been show to contain abundant P^S129^α-syn deposits, were obtained with a vibratome (HM 650 V Microm International), immunolabeled for P^S129^α-syn and then incubated with 3,3'-diaminobenzidine (DAB) or gold [35]. Ultrathin sections (60 nm) were analyzed with a JEOL 1200EX II electron microscope at 80 kV (Akishima, Japan).

### Antibodies

Mouse monoclonal anti-α-synD135 and anti-α-synY133 antibodies were generated essentially as previously described [2], with the peptides CGGEEGYQD and CGGPSEEGY, the corresponding synuclein sequences with three-residue extensions at the N-terminal end for coupling, as immunogens. These peptides were coupled to sheep anti-mouse IgG (Jackson ImmunoResearch Laboratories, West Grove, PA) with sulfo-EMCS (Molecular Biosciences, Boulder CO). Sera were then purified with Protein A-Sepharose and the antibodies were used at dilutions of 1:1000 (anti-α-synD135) or 1:700 (anti-α-synY133). We also used the following commercially available primary antibodies: rabbit polyclonal anti-ubiquitin (1:1000) from DAKO, rabbit monoclonal anti-P^S129^α-syn (1:2500 for electron microscopy, otherwise 1:5000) from Abcam; mouse monoclonal anti-P^S129^ α-syn (1:5000, WAKO); and rabbit polyclonal anti-full length α-syn (1:1000) from Affinity Biologicals. For immunohistochemistry, we used the following secondary antibodies: goat anti-rabbit or anti-mouse IgG conjugated to Alexa Fluor 488 (1:1000, Invitrogen), donkey anti-rabbit or anti-mouse IgG conjugated to Cy3 (1:500, Jackson).

### Mass spectrometry analysis (MS)

Sodium dodecyl sulfate (SDS)-soluble brain fractions [16] were run on an SDS polyacrylamide (15%) gel, which was then stained with Coomassie blue. Gel pieces containing the proteins of interest were destained and desiccated by incubation twice, for 20 min each, in 200 μl of 50 mM ammonium bicarbonate, 50% ethanol. Samples were then dried, incubated with modified trypsin (12.5 ng/μl) overnight at 37°C and stored at -80°C. The digested peptides were resuspended in 20 μl of 0.75% trifluoroacetic acid (TFA), 60% acetonitrile, 300 mg/ml lactic acid (solution A), loaded onto home-made titanium tips [36], equilibrated with solution A and washed with solution A supplemented with 0.1% TFA and 80% acetonitrile (ACN). Samples were eluted twice with 0.5% ammonium hydroxide and 0.5% piperidine. Eluted fractions were acidified with formic acid (FA) and dried in a Speedvac. Peptides were resuspended in 2% ACN/0.1% FA for LC-MS/MS analysis (liquid chromatography - MS). MS analysis was performed on an LTQ Orbitrap XL (Thermo Fisher Scientific) equipped with a nanoACQUITY system (Waters). Tandem MS was carried out in an information-dependent mode, in which each full MS analysis was followed by three MS/MS acquisitions, with peptides selected for collision-induced dissociation (CID), to generate tandem mass spectra. The results of the data searches were imported into Scaffold (Version 3_00_8 Proteome Software) for the validation of protein identification. Mascot 2.3 (Matrix Science) and SEQUEST in Proteome Discoverer v.1.2 were used for data searches against a database for the human and mouse α-syn forms, and spectra were validated by manual inspection.

### Production of recombinant proteins, *in vitro* phosphorylation assays and western blot analyses

Early stop codons were introduced into the sequence of wild-type α-syn, in the pT7.7 backbone, by single-site mutagenesis with complementary oligonucleotides (α-synD135, forward and reverse primers: gccttctgaggaagggtatcaagactaetaacctgaagcctaag, cttaggcttcaggttattagtcttgatacccttcctcagaaggc; α-synY133, forward and reverse primers: ctgaggaagggtattaatgactacgaacctgaagcctaag, cttaggcttcaggttcgtagtcattaatacccttcctcag). All constructs were verified by sequencing. The recombinant proteins were produced and phosphorylated by Polo-like kinase 3 (PLK3, Invitrogen) as previously described [37,38]. Recombinant proteins were resolved by SDS-PAGE in 4-12% polyacrylamide gels, electrotransferred to nitrocellulose membranes and probed with anti-α-synD135 (1:400), anti-α-synY133 (1:400) or anti-α-syn (1:1000, Affiniti) antibodies. Antibody binding was visualized by incubation with secondary anti-rabbit IgG (1:50000, Jackson Laboratories) and enhanced chemiluminescence (West Pico Chemiluminescent substrate, Pierce or West Dura Extended Duration Substrate, Thermoscientific) with a Kodak Image J Station 4000 MM.

### Statistical analyses

Unpaired two-tailed Student’s *t*-tests were used to compare colocalization scores and the proportion of double-labeled neuronal cell bodies or processes between mouse genotypes hA30Pα-syn+/+ and *parkin*-/-; hA30Pα-syn+/+. SAS software version 9.1 was used to analyze data from the behavioral study. The extension reflex and the footprint data were analyzed by repeated measures ANOVA, the factors used for the linear models being age and genotype. For the footprint analysis, the models included a linear, a quadratic and a cubic term and were adjusted for weight. Stepwise descending selection was used to eliminate nonsignificant terms to obtain the most parsimonious model; the difference from zero of each group was estimated with nested models. Two genotypes were considered to be significantly different if at least one of the linear, quadratic or cubic factors of the model differed between them. All of the differences observed concerned the cubic term. The *p*-values reported for each graph therefore correspond to the comparison of this term between groups. The final models were as follows:

(1) Symmetry of the hind limb extension reflex: wild-type = 0.03743.weight + 0.985 – 0.00239.age, hA30Pα-syn+/+ = 0.03743.weight + 1.0863 – 0.02742.age – 0.001548.age^2^ – 0.00003.age^3^, *parkin*+/-; hA30Pα-syn+/+ = 0.03743.weight + 0.9866 – 0.02692.age – 0.000889.age^2^ – 0.00001.age^3^, *parkin*-/-; hA30Pα-syn+/+ = 0.03743.weight + 1.0286 – 0.0143.age + 0.000664.age^2^ – 0.00000984.age^3^, *parkin*-/-; hA30Pα-syn+/- = 0.03743.weight + 0.9833, *parkin*-/- = 0.03743.weight + 1.0504.

(2) Amplitude of the hind limb extension reflex: wild-type = 0.1311.weight + 0.8324 – 0.2131.age + 0.005619.age^2^ – 0.00005.age^3^, hA30Pα-syn+/+ = 0.1311.weight – 0.00003.age^3^, *parkin*+/-; hA30Pα-syn+/+ = 0.1311.weight – 0.00002.age^3^, *parkin*-/-; hA30Pα-syn+/+ = 0.1311.weight + 1.2297 – 0.05299.age + 0.00021.age^2^, *parkin*-/-; hA30Pα-syn+/- = 0.1311.weight + 0.9174, *parkin*-/- = 0.1311.weight + 1.3363 – 0.00021.age^2^.

(3) Rotarod test: wild-type = 2.6585.weight + 110.04 – 1.41.age + 0.016.age^2^, hA30Pα-syn+/+ = 2.6585.weight + 138.88 – 5.42.age + 0.223.age^2^ – 0.00435.age^3^, *parkin*+/-; hA30Pα-syn+/+ = 2.6585.weight + 98.17 – 0.00108.age^3^, *parkin*-/-; hA30Pα-syn+/+ = 2.6585.weight + 112.46 – 0.00054.age^3^, *parkin*-/-; hA30Pα-syn+/- = 2.6585.weight + 107.98, *parkin*-/- = 2.6585.weight + 110.72.

(4) Length of forelimb stride: wild-type = 0.5815.weight + 5.1139 – 0.00000832.age^3^, hA30Pα-syn+/+ = 0.5815.weight + 4.6383 + 0.004034.age^2^ – 0.00015.age^3^, *parkin*+/-; hA30Pα-syn+/+ = 0.5815.weight + 5.3843 – 0.1668.age + 0.01054.age^2^ – 0.00019.age^3^, *parkin*-/-; hA30Pα-syn+/+ = 0.5815.weight + 5.5677 – 0.03991.age, *parkin*-/-; hA30Pα-syn+/- = 0.5815.weight + 5.0679 *parkin*-/- = 0.5815.weight + 5.0634.

## Results

### Parkin depletion delays the appearance of motor dysfunction in a dose-dependent manner in hA30Pα-syn mice

**Figure 1:**
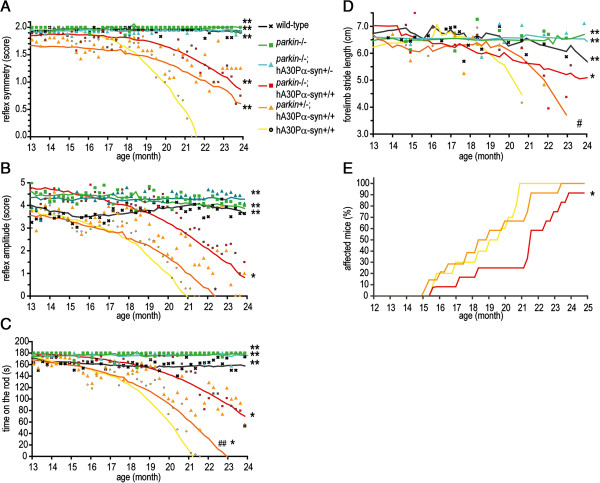
**Parkin deficiency delays the appearance of motor dysfunction in hA30Pα-syn mice in a dose-dependent manner.** Motor functions were assessed with the extension reflex **(A, B)**, the rotarod **(C)** and footprint **(D)** tests. For each test and for each genotype, the mean performance scores are presented as a function of age (*n* = 11–14 per genotype); the curves correspond to the weight-adjusted models used for the statistical comparisons. **(E)** The penetrance of the phenotype was calculated as the age at which the animals were unable to perform the rotarod test (Kaplan-Meier analysis). *; **: different from hA30Pα-syn+/+ mice with *p* < 0.01; *p* < 0.0001; #; ##: different from *parkin*-/-; hA30Pα-syn+/+ mice, with *p* < 0.05; *p* < 0.0001.

We previously reported that the loss of both alleles of *parkin* significantly delayed the onset of hindlimb extension reflex asymmetry and the decline of rotarod performance in hA30Pα-syn mice [16]. We evaluated the effects of a single dysfunctional *parkin* allele on disease-related changes in motor function in this model, by analyzing in more detail the behavior of hA30Pα-syn mice with no, one, or two dysfunctional *parkin* alleles during a longitudinal follow-up study. We assessed the performance of the animals in three behavioral tasks: the hindlimb extension reflex, analyzed here not only for loss of symmetry, but also for decline in amplitude, (2) the rotarod test and (3) the footprint test (Figure 1). The extension reflex data confirmed the progressive impairment previously reported in hA30Pα-syn mice (*p* < 0.0001 *versus* wild-type mice) and its significantly later onset in mice with two dysfunctional *parkin* alleles (*p* < 0.0001 for symmetry and *p* < 0.01 for amplitude *versus* hA30Pα-syn+/+; Figure 1A,B) [16]; a similar delay was observed for hA30Pα-syn with a single dysfunctional *parkin* knockout allele (*p* < 0.0001 for symmetry and *p* < 0.01 for amplitude *versus* hA30Pα-syn+/+). Similarly, the mean rotarod performances of hA30Pα-syn mice declined with age (*p* < 0.0001 *versus* wild-type mice; Figure 1C), and this loss of performance was delayed in the absence of functional *parkin* alleles (*p* < 0.01 *versus* hA30Pα-syn+/+); a significant, but less pronounced delay, was also observed with a single *parkin* knockout allele (*p* < 0.01 *versus* hA30Pα-syn+/+ and *p* < 0.0001 *versus parkin*-/-; hA30Pα-syn+/+). An analysis of gait characteristics in the footprint test confirmed these observations (Figure 1D): the mean length of forepaw strides decreased with age in hA30Pα-syn mice (*p* < 0.0001 *versus* wild-type mice); this decrease was delayed in mice with no functional *parkin* alleles (*p* < 0.01 *versus* hA30Pα-syn+/+) and, to a lesser extent, in hA30Pα-syn mice with a single functional *parkin* allele (*p* < 0.05 *versus parkin*-/-; hA30Pα-syn+/+).

Extension amplitude emerged as the earliest presymptomatic impairment in each group, followed by a decline in performance of the rotarod task, asymmetry in hindlimb extension and a decrease in stride length (Table 1). In each task, hA30Pα-syn mice with a single dysfunctional *parkin* allele had an intermediate phenotype, between that of hA30Pα-syn mice with no dysfunctional allele and that of hA30Pα-syn mice with two dysfunctional *parkin* alleles. The absence of Parkin lowered the penetrance of the phenotype, defined as the proportion of animals unable to perform the rotarod task at a given age (Figure 1E), whereas the partial depletion of Parkin had no such effect.

**Table 1:** Age by which a 50% decrease in the mean test performance of each group had occurred, or a 25% decrease in the case of the footprint test, within a confidence interval of one week, determined from the mathematical models established

	**Amplitude of extension reflex score < 2.5**	**Rotarod time on rod < 90 s**	**Symmetry of extension reflex score < 1**	**Stride length < 5 cm**
hA30Pα-syn+/+	18.1 ± 0.2 months	19.3 ± 0.2 months	20.0 ± 0.2 months	20.7 ± 0.2 months
*parkin*+/-; hA30Pα-syn+/+	18.4 ± 0.2 months	20.2 ± 0.2 months	22.3 ± 0.2 months	23.0 ± 0.2 months
*parkin*-/-; hA30Pα-syn+/+	21.0 ± 0.2 months	23.2 ± 0.2 months	23.4 ± 0.2 months	> 24 months

### Truncated α-syn species found in LB accumulate in symptomatic hA30Pα-syn mice in the presence and absence of Parkin

**Figure 2:**
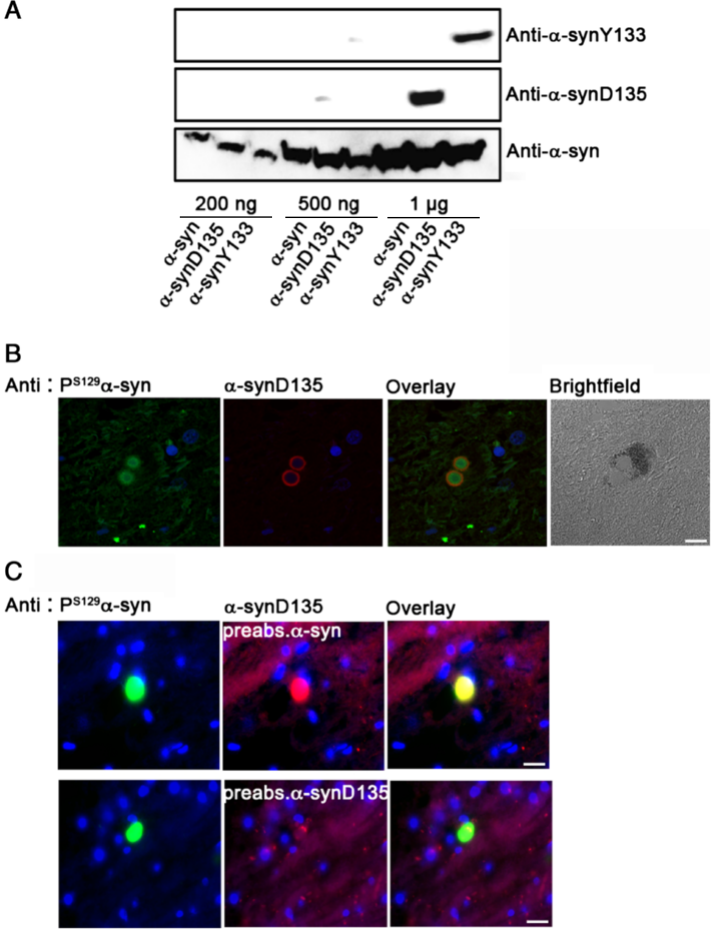
**A new antibody reveals the presence of α-synD135 in the LB of PD cases. (A)** Immunoblot analysis with recently developed antibodies directed against recombinant α-syn and its truncated versions. **(B)** Colocalization analysis of α-synD135 and P^S129^α-syn immunoreactivities within LB, in a melanized dopaminergic neuron (brightfield image) in the *substantia nigra* of a representative PD patient. **(C)** Control experiment showing that the staining for α-synD135 in LB is abolished when the antibody is absorbed with the truncated, but not with the full-length, recombinant α-syn protein before immunohistochemistry. The blue signal corresponds to nuclear DNA stained with DAPI. Scale bar: 10 μm.

We explored the mechanisms underlying the protection associated with Parkin deficiency, by comparing the accumulation of pathological α-syn species in diseased hA30Pα-syn mice with functional *parkin*, and in hA30Pα-syn mice with no functional *parkin* alleles. Enrichment in α-synD135 or α-synY133 has been reported in the brains of PD patients studied by biochemical techniques [2]. We investigated the accumulation of these species in hA30Pα-syn mice, using recently developed antibodies that we validated by western blotting and immunohistochemistry. These antibodies could not detect amounts below 1 μg of their cognate recombinant protein, thus limiting their value for biochemical studies (Figure 2A). However, the analysis of more than 50 LB in *substantia nigra* sections from three cases of sporadic PD revealed immunostaining for α-synD135 in more than 90% of these inclusions, detected with P^S129^α-syn antibodies (Figure 2B). No such staining was observed when the anti-α-synD135 antibody was preabsorbed with α-synD135, whereas prior incubation with the full-length α-syn did not abolish the signal (Figure 2C). By contrast, the preabsorption experiment revealed nonspecific staining with the antibody against α-synY133 (data not shown). This antibody was therefore not used in subsequent experiments.

**Figure 3:**
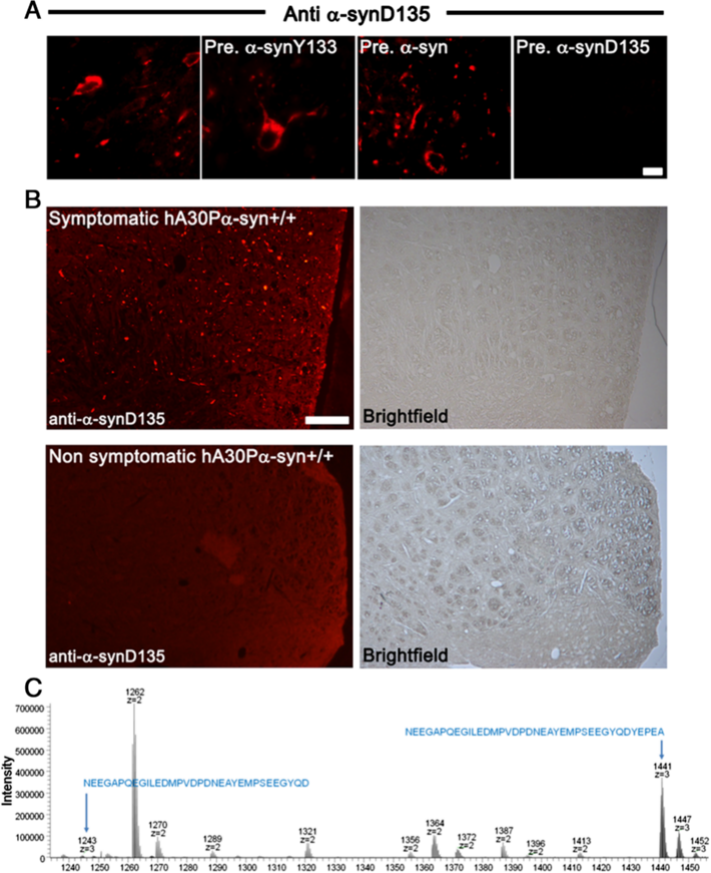
**α-SynD135 accumulates specifically in the central nervous system of symptomatic hA30Pα-syn mice, regardless of *parkin *expression status. (A)** Micrographs illustrating specific immunoreactivity in coronal brainstem sections from a representative symptomatic hA30Pα-syn mouse with functional *parkin* alleles, stained with anti-α-synD135 antibody before (left) and after absorption with recombinant α-synY133, full-length α-syn or α-synD135. Scale bars: 25 μm. **(B)** Comparative staining for α-synD135 in the brainstem of representative symptomatic and healthy hA30Pα-syn mice with functional *parkin* alleles; similar results were obtained in hA30Pα-syn mice with no functional *parkin* alleles. Scale bars: 100 μm. **(C)** Representative spectrum illustrating the detection, by MS, of the C-terminal fragment of full-length α-syn (mass/charge = 1441 with z = 3) and α-synD135 (mass/charge = 1243 with z = 3) in the SDS-soluble fraction of proteins from brain lysates from hA30Pα-syn+/+ mice; peptides (sequence indicated in blue) were fragmented and unambiguously identified by tandem MS.

We then investigated the accumulation of α-synD135 species in hA30Pα-syn mice with functional or dysfunctional *parkin*. In the brainstem of symptomatic animals, the anti-α-synD135 antibody labeled numerous normally shaped or swollen processes and cell bodies (Figure 3A). This labeling was abolished by prior absorption with recombinant α-synD135 (Figure 3A), and was not detected in the brains of healthy hA30Pα-syn mice, regardless of their expression status for *parkin*, or in non transgenic animals (Figure 3B and data not shown). Qualitative comparisons of symptomatic hA30Pα-syn mice and hA30Pα-syn mice with no functional *parkin* alleles revealed no obvious differences attributable to Parkin deficiency in terms of the type of structures labeled (processes *versus* cell bodies) or the density of deposits (data not shown). MS analyses of brain lysates from symptomatic mice unambiguously identified the presence of α-syn and α-synD135 in both hA30Pα-syn mice and hA30Pα-syn mice lacking Parkin (representative spectrum shown in Figure 3C). By contrast, the signal potentially corresponding to α-synY133 was weak in all cases, precluding clear identification of this species (Table 2).

**Table 2:** **Summary of the α-syn species detected by MS in brain lysates from two hA30Pα-syn mice with functional *parkin *** **(column # 1–2) and three hA30Pα-syn mice lacking both *parkin *** **alleles (column # 3–5)**

		**hA30Pα-syn+/+**		***parkin*-/-; hA30Pα-syn+/+**		
		# 1	# 2	# 3	# 4	# 5
Human	α-syn	+ +	+ +	+ +	+ +	+ +
	α-synD135	+ +	+ +	-	+	+ +
	α-synY133	-	-	-	-	-
Mouse	α-syn	+ +	+ +	+	+	+ +
	α-synD135	-	-	-	-	-
	α-synY133	-	-	-	-	-

-: not detected; +: identified with a probability between 80% and 94%; ++: identified with a probability >95%.

### The α-synD135 species deposited are preferentially associated with P^S129^α-syn in the presence and absence of Parkin

**Figure 4:**
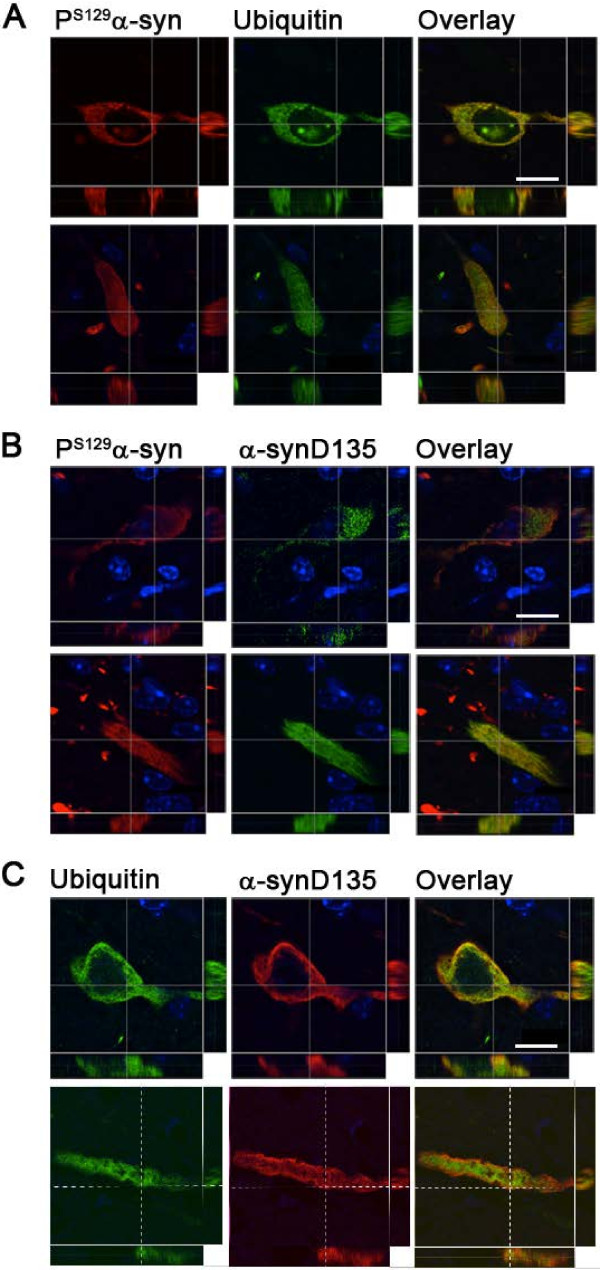
**Ubiquitin, and α-synD135 colocalize with P**^**S129**^**α-syn in hA30Pα-syn mice, regardless of *parkin *expression status.** Analysis by confocal microscopy, showing the degree of colocalization between **(A)** P^S129^α-syn and ubiquitin, **(B)** P^S129^α-syn and α-synD135, and **(C)** ubiquitin and α-synD135, in coronal brain sections from a representative symptomatic hA30Pα-syn mouse; similar observations were made in hA30Pα-syn mice with two or no functional *parkin* alleles (bregma -5.8). The projection stacks correspond to sections of single immunostained cell bodies (upper panels) and processes (lower panels) following the horizontal or vertical dashed line in the Z-stack. Scale bars = 10 μm.

**Figure 5:**
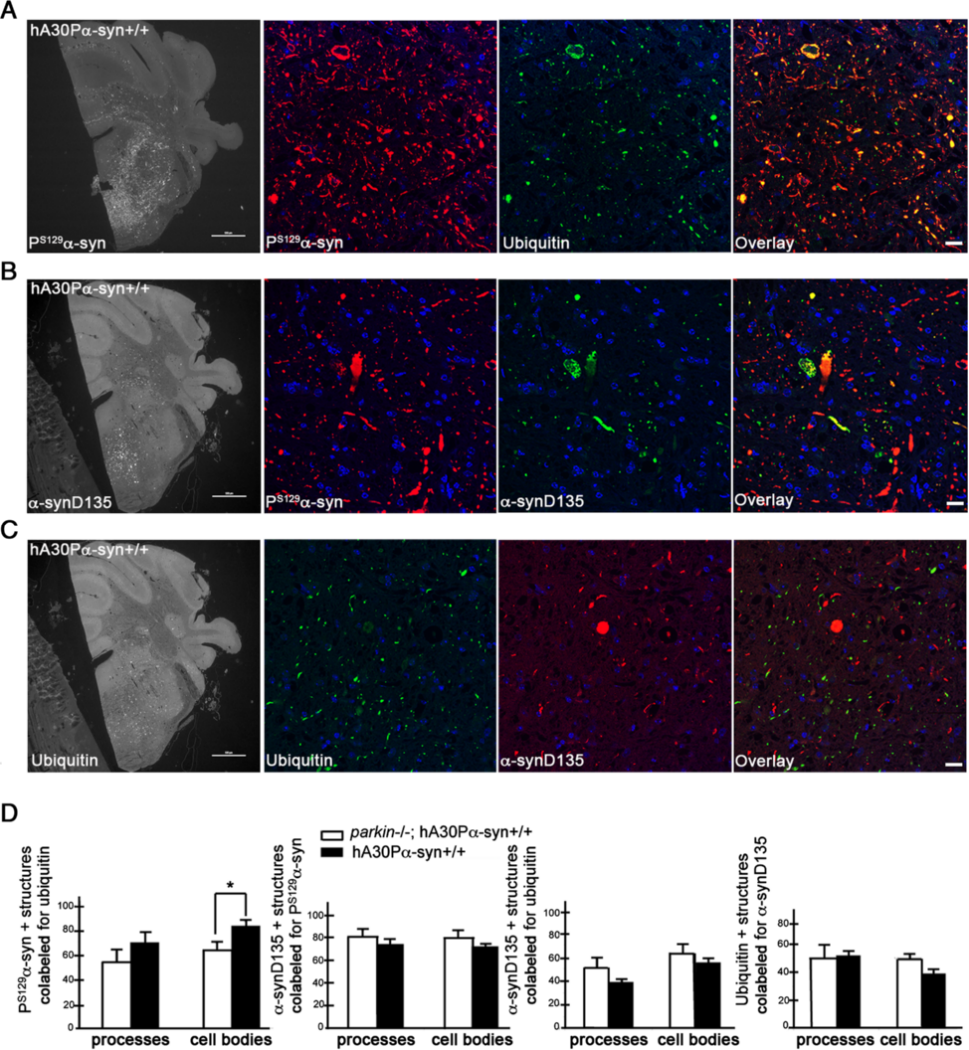
**Ubiquitin and α-synD135 associate preferentially with P**^**S129**^**α-syn deposits in hA30Pα-syn mice, regardless of *parkin *expression status.** Epifluorescence microscopy illustrating the association between **(A)** P^S129^α-syn and ubiquitin, **(B)** P^S129^α-syn and α-synD135, and **(C)** ubiquitin and α-synD135, in hemi-brain sections from a representative symptomatic hA30Pα-syn mouse with normal *parkin* alleles (bregma -5.8). In blue: nuclear DNA stained with DAPI. The left micrographs illustrate representative regions immunostained for each marker, at low magnification (Scale bars: 1000 μm), and the associated triad of micrographs are higher magnifications of areas within these regions (Scale bars: 20 μm). **(D)** Quantitative analysis of the proportion of colabeled neuronal cell bodies and processes in hA30Pα-syn with two or no functional *parkin* alleles. (*n* = 6–10).

In our previous study, we reported that ubiquitin immunoreactivity invariably colocalized with P^S129^α-syn staining in symptomatic hA30Pα-syn mice. In addition, the P^S129^α-syn deposits in neuronal cell bodies and processes were less frequently associated with ubiquitin in mice with no functional *parkin* alleles than in mice with functional *parkin*[16]. Confocal analysis of a larger group of animals in this study confirmed the colocalization of ubiquitin staining and P^S129^α-syn deposits in symptomatic hA30Pα-syn mice, regardless of their *parkin* status (Figure 4A, representative Pearson’s correlation coefficient: 0.8 ± 0.07; Figure 5A). We also confirmed that the proportion of P^S129^α-syn-positive cell bodies associated with ubiquitin staining was lower in mice with no functional *parkin* alleles than in mice with functional *parkin* (Figure 5D, left graph). However, this difference was not significant for neuronal processes.

We investigated whether α-synD135 was phosphorylated or ubiquitylated and whether there were differences in these modifications associated with Parkin deficiency, by performing double immunofluorescence staining on brain sections from symptomatic hA30Pα-syn mice with functional *parkin* or with no functional *parkin* alleles. Staining for α-synD135 was significantly less abundant than staining for P^S129^α-syn (Figure 5B) and ubiquitin (Figure 5C), in mice of both genotypes. Consistent with the observations in LB, most α-synD135 immunoreactivity was found to be associated with P^S129^α-syn-positive deposits (Figure 5B and Figure 5D, second graph from the left). However, it often did not cover the entire P^S129^α-syn-positive structure within a particular neuronal cell body or process, or extended beyond it (Figure 5B). The degree of colocalization within regions displaying both types of immunoreactivity was high, regardless of *parkin* expression status (Figure 4B, representative Pearson’s correlation coefficient: 0.9 ± 0.04). α-SynD135 and ubiquitin colocalized in some neurons, but were also observed independently of each other in approximately 50% of the neuronal cell bodies and processes in symptomatic hA30Pα-syn mice, regardless of *parkin* expression status (Figure 4C, representative Pearson’s correlation coefficients: 0.8 ± 0.03; Figure 5C and D, graphs on the right).

**Figure 6:**
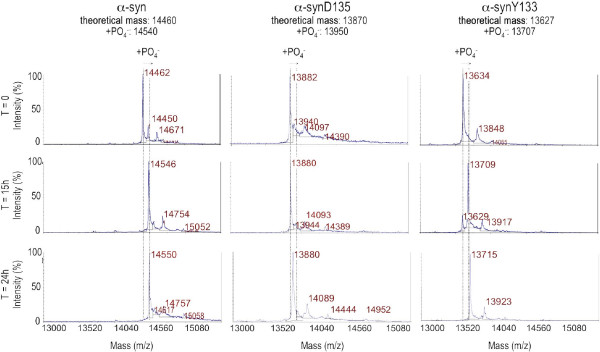
**PLK3 does not phosphorylate α-synD135 efficiently *in vitro*.** MALDI-TOF time-point analyses of the phosphorylation of recombinant α-syn, α-synD135 and α-synY133 by PLK3: the mass increase of approximately 80 Da corresponds to the addition of one phosphate group.

We investigated the effect of truncation on α-syn phosphorylation at S129, by performing *in vitro* phosphorylation assays with PLK3, which is known to phosphorylate α-syn efficiently and specifically at S129 [37,39]. After 15 h, the entire pool of full-length α-syn was phosphorylated by PLK3, whereas the α-synY133 variant was only partially converted and phosphorylated α-synD135 was undetectable after 15 h (Figure 6).

### P^S129^α-syn accumulates as fibrillar species in symptomatic hA30Pα-syn mice with and without Parkin

**Figure 7:**
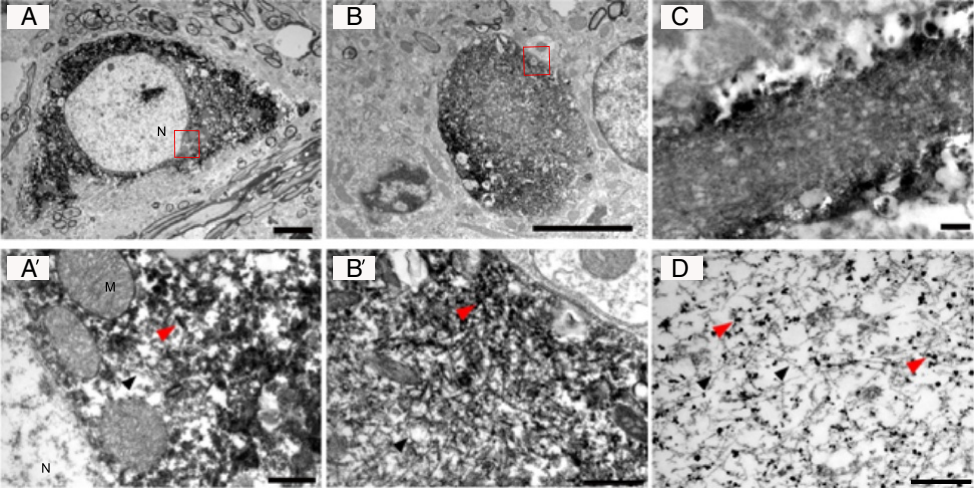
**P**^**S129**^**α-syn accumulates in fibrillar deposits in hA30Pα-syn mice.** Representative electron micrographs illustrating a cell body **(A)** and a process sectioned transversally **(B)** or longitudinally **(C)** and immunostained with P^S129^α-syn antibodies and secondary antibodies coupled to horseradish peroxidase from a representative symptomatic hA30Pα-syn mouse expressing *parkin*. **(A’-B’)** are higher magnifications of the framed regions in **(A)** and **(B)**. **(D)** Nanogold staining of a P^S129^α-syn deposit within a neuronal process. Arrowheads indicate fibrils with (red) or without (black) associated P^S129^α-syn staining; N (nucleus), M (mitochondria). Similar results were obtained in hA30Pα-syn mice with two or no functional *parkin* alleles. Scale bars: 4 μm in **(A)** and **(B)**, and 0.5 μm in **(A’)**, **(B’)**, **(C)** and **(D)**.

We used electron microscopy coupled to anti-P^S129^α-syn-specific immunostaining to investigate the nature of the deposits in regions of the brainstem in which a massive accumulation of this protein was observed and to explore potential differences associated with Parkin deficiency. Abundant fibrils loosely associated and diffusely distributed throughout the cytosol of neurons or in neuronal processes were observed with secondary antibodies conjugated to horseradish peroxidase, in mice of both genotypes (representative images shown in Figure 7A-C). There were no dense inclusions. The fibrils were generally more densely packed in the neuronal processes than in the cell bodies, and were, in some cases, confined to the proximity of the plasma membrane (Figure 7C). These fibrils were approximately 10–15 nm wide, as estimated for deposits immunostained with secondary antibodies coupled to gold beads (Figure 7D). Similar configurations were found in hA30Pα-syn mice with functional *parkin* and with no functional *parkin* alleles.

## Discussion

This study confirms and extends our previous work showing that the loss of both *parkin* alleles significantly delays sensorimotor impairment related to the progression of α-synucleinopathy in hA30Pα-syn mice [16]. We report here the results of a more complete comparative behavioral longitudinal follow-up study of hA30Pα-syn mice with two functional *parkin* alleles, or with the loss of one or both *parkin* alleles. In addition to the rotarod task and the analysis of hindlimb reflex symmetry, we used the hindlimb reflex amplitude and footprint tests to evaluate the behavior of the animals. Strikingly, this follow-up not only confirmed our previous results, but also revealed a protective effect of the loss of a single *parkin* allele. In addition, Parkin depletion had a clear dose-dependent effect on performance in the rotarod task, with different performance curves for hA30Pα-syn mice with two, one or no functional *parkin* alleles.

The loss of *parkin* expression delayed sensorimotor impairment in hA30Pα-syn mice, but only slightly modified neuropathological presentation in end-stage disease. The extent and intensities of the immunoreactivities against ubiquitin, P^S129^α-syn and α-syn truncated at D135 were generally similar in hA30Pα-syn mice with and without *parkin* expression. Consistent with the current hypothesis that α-syn phosphorylated at S129 plays a key role in the neuropathological process, P^S129^α-syn was deposited as loose fibrils throughout the brainstem and the spinal cord in affected animals only, regardless of *parkin* expression status. In hA30Pα-syn mice of both *parkin* genotypes, some of the P^S129^α-syn-positive deposits colocalized with immunostaining for ubiquitin or α-synD135, which were significantly less widely distributed than the P^S129^α-syn staining, consistent with observations in human brains affected by α-synucleinopathy. Ubiquitin and α-synD135 were mostly found concomitantly with P^S129^α-syn, indicating that ubiquitylation and C-terminal truncation at D135 are secondary PTM. As reported in our previous study [16], the proportion of P^S129^α-syn deposits in neuronal cell bodies associated with ubiquitin was lower in hA30Pα-syn mice lacking both *parkin* alleles than in hA30Pα-syn mice with functional *parkin* alleles, possibly indicating less advanced α-synucleinopathy. By contrast, *parkin* dysfunction was not associated with any significant difference in the proportion of P^S129^α-syn deposits costained for α-synD135, or in the degree of colocalization between α-synD135- and ubiquitin-immunoreactive protein deposits in the neuronal somata or processes of hA30Pα-syn mice. It is widely accepted that ubiquitylation is primarily associated with P^S129^α-syn in human α-synucleinopathies, but less is known about the crosstalk between S129 phosphorylation and C-terminal truncation. Anderson *et al*. [2] reported that all peptides corresponding to the α-synY133 fragment identified by MS were phosphorylated at S129 in LB fractions [2]. We found that recombinant α-synY133 and α-synD135 proteins were less well phosphorylated by PLK3 than the full-length protein *in vitro*[37,39], suggesting that C-terminal truncation does indeed occur once the protein has been phosphorylated. However, the partial dissociation between immunostaining for α-synD135 and that for P^S129^α-syn often observed in the double-labeled neuronal cell bodies and processes of hA30Pα-syn mice, regardless of *parkin* expression, suggests that α-syn truncation at D135 may also arise independently of phosphorylation on S129. Alternatively, there may be interference between the two types of immunohistochemical staining, due to the physical proximity of the PTM examined: it is not unlikely, for example, that a deposit of P^S129^α-syn with a high proportion of truncated protein is less efficiently recognized by antibodies specific for the phosphorylated species.

There is some debate about whether the ubiquitylation of α-syn constitutes a proteasomal degradation signal [1], but proteasome dysfunction, which is thought to occur during the progression of α-synucleinopathy [29,40,41], may generate C-terminally cleaved α-syn fragments [12,42]. However, the random nature of the association between the patterns of immunostaining for ubiquitin and α-synD135 in our study is not consistent with ubiquitylation playing a role in the generation of such fragments.

Despite the mitigation of the preclinical α-syn-related phenotype revealed by our longitudinal behavioral follow-up study, the partial depletion of Parkin did not delay overt manifestation of the neurological phenotype in hA30Pα-syn mice. The lack of impact of Parkin deficiency on disease penetrance and progression reported in hA53Tα-syn mice [31], therefore does not exclude a moderate modifier effect, as the study concerned was based on late-stage neurological phenotypes and single-point analyses of specific behavioral measures. Alternatively, the absence of interaction between Parkin and α-synA53T may reflect different effects of Parkin deficiency on the pathological alterations caused by different α-syn variants [31]. However, a recent *in vitro* study reported the rescue of cortical neurons from the harmful effects of α-synA53T toxicity by siRNA-mediated downregulation of the *parkin* gene, supporting the notion that there is a beneficial modifier effect associated with this condition [43].

## Conclusion

In conclusion, we confirm here that Parkin depletion modifies α-syn-induced neurodegeneration in hA30Pα-syn mice. The loss of a single functional *parkin* allele was sufficient to delay the appearance of preclinical signs of motor dysfunction in this model, albeit to a lesser extent than observed with the loss of both alleles, with no effect on appearance of overt disease signs. An analysis of the α-syn protein deposits revealed possible interplay between PTM: ubiquitylation and truncation at D135 were associated preferentially with P^S129^α-syn, whereas ubiquitylation and α-synD135 were found both independently and in association. The distribution and composition of the P^S129^α-syn deposits were similar in the presence and absence of Parkin in the end stage of disease, suggesting that a loss of Parkin function does not fundamentally alter the nature of events leading to protein accumulation in this mouse model. A detailed quantitative investigation of the proteins, signaling and degradation pathways thought to regulate α-syn-dependent neurodegeneration during the preclinical and early symptomatic stages of the disease might shed light on the mechanisms underlying the neuroprotection associated with Parkin deficiency in future studies.

## Abbreviations

PD: Parkinson’s disease; LB: Lewy bodies; α-syn: α-synuclein; PTM: Posttranslational modifications; PS129α-syn: α-syn phosphorylated at S129; hA30α-syn: Mouse model producing the pathogenic human A30P variant of α-syn; α-synD135 and α-synY133: α-syn truncated at D135 and Y133; hA30Pα-syn: Human A30P variant of α-syn; MS: Mass spectrometry; PLK3: Polo-like kinase 3; SDS: Sodium dodecyl sulfate; PBS: Phosphate-buffered saline; BSA: Bovine serum albumin; NGS: Newborn goat serum; PFA: Paraformaldehyde; TFA: Trifluoroacetic acid; ACN: Acetonitrile; FA: Formic acid.

## Competing interests

None of the authors has any conflict of interest to report.

## Authors’ contributions

MF designed, performed the behavioral study and the *in vitro* experiments, analyzed the data and wrote the manuscript. AR designed, performed the immunohistological characterization, experiments, analyzed the data and wrote the manuscript. JG genotyped the animals and participated in the behavioral analyses. MPM performed electron microscopy experiments. PB and STM analyzed the behavioral data. HAL provided recombinant α-syn, participated in the design of the *in vitro* experiments and helped draft the manuscript. JPA helped draft the manuscript. JPA, RB and JH generated the antibodies against truncated α-syn. AB participated in study design and reviewed the manuscript. OC designed the study, analyzed the data and wrote the manuscript. All authors have read and approved the manuscript.
